# Astrocyte L-Lactate Signaling in the ACC Regulates Visceral Pain Aversive Memory in Rats

**DOI:** 10.3390/cells12010026

**Published:** 2022-12-21

**Authors:** Zafar Iqbal, Shu Liu, Zhuogui Lei, Aruna Surendran Ramkrishnan, Mastura Akter, Ying Li

**Affiliations:** 1 Department of Neuroscience, College of Veterinary Medicine and Life Sciences, City University of Hong Kong, Hong Kong 999077, China; 2 Department of Biomedical Sciences, College of Veterinary Medicine and Life Sciences, City University of Hong Kong, Hong Kong 999077, China; 3 Centre for Regenerative Medicine and Health, Hong Kong Institute of Science & Innovation, Chinese Academy of Sciences, Hong Kong 999077, China; 4 Centre for Biosystems, Neuroscience, and Nanotechnology, City University of Hong Kong, Hong Kong 999077, China

**Keywords:** astrocyte, lactate, anterior cingulate cortex (ACC), optogenetic, chemogenetic, colorectal distension, conditioned place avoidance (CPA), aversion memory

## Abstract

Pain involves both sensory and affective elements. An aspect of the affective dimension of pain is its sustained unpleasantness, characterized by emotional feelings. Pain results from interactions between memory, attentional, and affective brain circuitry, and it has attracted enormous interest in pain research. However, the brain targets and signaling mechanism involved in pain remain elusive. Using a conditioned place avoidance (CPA) paradigm, we show that colorectal distention (CRD magnitude ≤ 35 mmHg, a subthreshold for pain) paired with a distinct environment can cause significant aversion to a location associated with pain-related insults in rats. We show a substantial increase in the L-lactate concentration in the anterior cingulate cortex (ACC) following CPA training. Local exogenous infusion of lactate into the ACC enhances aversive memory and induces the expression of the memory-related plasticity genes pCREB, CREB, and Erk1/2. The pharmacological experiments revealed that the glycogen phosphorylation inhibitor 1,4-dideoxy-1,4-imino-D-arabinitol (DAB) impairs memory consolidation. Furthermore, short-term Gi pathway activation of ACC astrocytes before CPA training significantly decreases the lactate level and suppresses pain-related aversive learning. The effects were reversed by the local infusion of lactate into the ACC. Our study demonstrates that lactate is released from astrocytes in vivo following visceral pain-related aversive learning and memory retrieval and induces the expression of the plasticity-related immediate early genes CREB, pCREB, and Erk1/2 in the ACC. Chronic visceral pain is an important factor in the pathophysiology of irritable bowel syndrome (IBS). The current study provides evidence that astrocytic activity in the ACC is required for visceral pain-related aversive learning and memory.

## 1. Introduction

Pain is a conscious subjective experience that is most often invoked by nociceptive stimulation [1] and does not always require tissue damage. Pain involves both sensory and affective elements. The anterior cingulate cortex (ACC), which receives input from limbic regions, such as the thalamus, hippocampus and amygdala, is essential to the control of selective attention and working memory [2]. The discovery of the ACC as the key brain area for modulating the sensory and affective aspects of visceral pain is arguably an important advancement in the field of brain targets of chronic pain [2,3,4]. 

We identified ACC sensitization and synaptic plasticity in a visceral hypersensitivity (VH) rat model in which rats were sensitized to chicken egg albumin [5,6]. ACC hypersensitivity to colorectal distension has been shown to last up to 7 weeks after the initiation of colonic anaphylaxis and is independent of mucosal inflammation, suggesting that the condition may be mediated by a mechanism for triggering pain memory [7,8]. The VH rats showed ACC circuitry desynchronization and enhanced visceral pain sensation [5,6,9,10]. 

The clinical connection between visceral pain and increased incidences of depression, social anxiety and other cognitive disorders has been well established [11,12]. One aspect of the affective dimension of pain is the sustained unpleasantness of pain, characterized by emotional feelings, such as fear and distress [13]. Using colorectal distention (CRD, pseudaffective reflex [14]) with the conditioned place avoidance (CPA) paradigm, our published data show that when CRD (magnitude ≤ 35 mmHg, a subthreshold for pain perception) was paired with a distinct environment in the conditioning apparatus, the rats spent significantly less time in this compartment on the post-conditioning test days, suggesting that rats experienced significant aversion to a location associated with pain-related insults [15,16]. This observation suggests that noxious visceral stimuli have motivational power and can support associative learning. 

Astrocytes, a glial cell type, can sense and modify synaptic activity at the surrounding synapses by releasing active substances and have been found to be involved in the modulation of various neuronal circuits [17,18]. Astrocytes support neuronal functions by regulating the flow of extracellular ions and neurotransmitters and by providing neuronal energy substrates such as lactate by means of their astrocytic processes [10,18,19,20]. Given that glutamate uptake stimulates the glycolysis of astrocytes to produce lactic acid, which is then transported to neurons as fuel, this suggests that glycolysis in astrocytes is dominant, while the oxidative metabolism of lactic acid in neurons is dominant [21]. 

Astrocytes, rather than neurons, store glycogen [19,21] that can be swiftly mobilized and directed to the glycolytic pathway to produce L-lactate. Studies in humans and animals have detected activity-dependent lactate release in activated brain areas during sensory and cognitive stimulation [22,23]. Our published data show that the optogenetic activation of astrocytes in the ACC elicits lactate release, improves decision making in the physiological state and rescues the impaired decision making in rats with chronic visceral pain [10]. The activation of astrocytes rescues ACC synaptic LTP and repairs impaired spike phase locking in rats with chronic visceral pain [10]. Given the important role of astrocytes in modulating pain, we tested our hypothesis that during pain aversive learning and memory recall, in response to noxious afferent stimulation, glutamate released from ACC neurons activates astrocytes and the astrocyte neuron lactate shuttle (ANLS) is involved in modulating pain-related aversive behavior. The active neurons particularly feed on the lactate generated by astrocytes from glucose and glycogen. In this study, we determined the lactate concentration in ACC areas following conditioned place avoidance (CPA) training, combined with various astrocytic manipulations. We showed that the exogenous infusion of lactate into the ACC enhances aversive memory and induces the expression of the memory-related plasticity genes CREB/pCREB and Erk1/2. We observed that blocking lactate production by the local infusion of the glycogen phosphorylation inhibitor 1,4-dideoxy-1,4-imino-D-arabinitol (DAB) [24,25] to inhibit glycogenolysis decreased the lactate level and impaired aversive memory. 

To understand whether normal astrocytic activity is necessary for the ACC to regulate aversive behavior, we manipulated ACC astrocytic activity through Gi pathway activation via hM4Di receptor activation during memory acquisition. We show that short-term chemogenetic Gi activation of ACC astrocytes blocked aversive memory retrieval after the initial CPA learning. The effects were restored by local exogenous lactate infusion. 

We provide evidence of an increased level of astrocyte-released lactate in the brain following pain-related aversive learning, which may play a critical role in regulating aversive memory retrieval and inducing the expression of the plasticity-related immediate early genes CREB/pCREB and Erk1/2.

## 2. Materials and Methods

### 2.1. Animal Experiments

All experimental protocols involving the use of animals were carried out on adult male Sprague–Dawley rats weighing approximately 250–300 g. They were kept in individual cages and maintained on a 12:12 light and dark cycle, with a constant room temperature of 25 °C. All rats were allowed ad libitum 24 h access to food chow and water. The animal studies were performed in accordance with the guidelines laid down by the Committee on the Use and Care of Animals, Department of Health, Government of Hong Kong SAR, Control of Experiments Ordinance (Cap. 340) and license to Conduct Experiments Reference No. 21–56 in DH/HT&A/8/2/5 Pt. 5; No. 21–50 in DH/HT&A/8/2/5 Pt.5; and Rev(19–177) in DH/HT&A/8/2/5 Pt. 1. The approvals for the “Ethical Review of Research Experiments Involving Animal Subjects” were granted by the Animal Research Ethics Sub-Committee, City University of Hong Kong (reference no. A-0215 and A-0513).

### 2.2. Cannula Implantation

Chronic cannulation is performed in the ACC for studies involving the infusion of drugs. The rats were anesthetized with a mixture of ketamine (65 mg/kg) and xylazine (7.5 mg/kg). Small holes were made with a drill machine on the skull surface according to the following coordinates: AP = +2.8 mm, ML = ±0.8 mm and DV = 2.8 mm from the skull surface. The 26-gauge stainless-steel guide cannulae were bilaterally positioned in the ACC region. The guide cannulae were fixed to the skull with the help of screws and dental cement (Megadental, Budingen, Germany). Dummy cannulae 0.5 mm longer than the guide cannulae were inserted into the guide cannulae to prevent blockage and to reduce the risk of infection. The rats were provided with one week to recover before starting the experimental protocols.

### 2.3. Drugs Administration

The following drugs were used and purchased from Sigma: 1,4-dideoxy-1,4-imino-D-arabinitol hydrochloride (DAB; Sigma-Aldrich, St. Louis, MO, USA; catalog no. D1542) and sodium L-lactate (Sigma-Aldrich, St. Louis, MO, USA; catalog no. L7022). The DAB (300 µM; 25) and sodium L-lactate (10 mM; 19) were mixed in 0.9% saline or aCSF. The drugs were mixed together and injected locally 15 min before the conditioning training, where appropriate, and infusion was performed at 1 µL per each ACC side. The infusion needles extended 0.5 mm beyond the guide cannula. The injection flow rate was kept at a rate of 0.333 mL/min [10], with an infusion pump (World Precision Instruments, Sarasota, FL, USA). The micro-injection needle was kept in place for an additional five minutes following the injection to allow for the complete dispersion of the solution. Clozapine-N-oxide hydrochloride (CNO; 1 mg/kg of BW; MedChemExpress (MCE), Monmouth Junction, NJ, USA; catalog no. HY-17366A) was dissolved in DMSO and then diluted in 0.9% saline to yield a final DMSO concentration of 0.5%. The saline solution for the control injections also consisted of 0.5% DMSO.

### 2.4. Optogenetic Manipulation of ACC Astrocytes

For the optogenetic activation of the ACC astrocytes, an AAV8-gfaABC1D-hChR2(H134R)-eGFP (5.5 × 10^12^, 0.45 µL; Taitool Bioscience, Shanghai, China) was injected bilaterally into the ACC (AP = +2.8 mm, ML = ±0.8 mm and DV = −2.8 mm from the surface of the skull). The flow rate of the injection was kept at a rate of 0.1 µL/min, and it was controlled via a microinjection pump (World Precision Instruments, Sarasota, FL, USA) using a 10 µl Hamilton syringe with a 33 G injection cannula. The needle was kept in the target site for another 5 min to allow for the proper diffusion of the virus and then slowly withdrawn. After the viral injection, the rats received bilateral surgical implantation of the chronic fiber optic cannula at 0.3 mm above the injection site and rostrally angled at 10^0^ toward the injection site (core diameter = 200 µm and numerical aperture = 0.39 NA; Thorlabs, USA). The control group was kept without any virus but still implanted with a fiberoptic cannula to deliver the blue light into the ACC.

For the optical stimulation of the ACC astrocytes, a blue light source with a pulse of 45 ms and a 20 Hz frequency was used [26]. The control group without virus only received the blue light, with the same parameters. These parameters were programmed using a waveform generator (Model AFG2021-SC; Tektronix, Beaverton, OR, USA) that was connected to a blue laser source, depending on the nature of the experiment (CNI Laser, Changchun, China). The rats received the blue light (3 min ON followed by 3 min OFF) for 45 min of conditioning training in the conditioning compartment and the last 5 min with the light off through a fiberoptic cable connected to a 1 × 2 intensity division fiberoptic rotary joint (Doric Lenses Inc., Rue Franquet, QC, Canada).

### 2.5. Chemogenetic Manipulation of ACC Astrocytes

The rats were anesthetized with a mixture of ketamine (65 mg/kg) and xylazine (7.5 mg/kg) and were placed on a stereotaxic apparatus (Kopf Instruments, LA, California, USA). The skull was exposed via incision, and an AAV5-GFAP-hM4D(Gi)-mCherry (5.5 × 10^12^, 0.45 µL; Addgene, Watertown, MA, USA; 50479-AAV5) was injected bilaterally into the ACC (AP = +2.8 mm, ML = ±0.8 mm and DV = 2.8 mm from the skull surface) using a 33 G Hamilton syringe. The injection volume and flow rate (0.1 µL/min) were controlled via an injection pump (Worlds Precision Instruments, Sarasota, FL, USA). The rats were kept undisturbed for three weeks after the virus injection to recover and to allow for gene expression. After three weeks, the rats underwent training and were divided into CNO-injected, saline-injected and control-CNO groups. Clozapine N-oxide hydrochloride (CNO; 1 mg/kg of BW; i.p.) was injected 30 min before the start of the conditioning training. The chosen low dosage of CNO did not induce any behavioral signs of seizure activity.

### 2.6. Behavioral Paradigm

Rats from the different experimental groups were randomly allocated to cages, and the researcher was blind to the treatment groups. All experiments were conducted under the same experimental conditions to prevent any bias arising from an experiment. The number of animals used in each experiment is specified in the figure legends.

Using a visceral pain assay that combines colorectal distension (CRD) with conditioned place avoidance (CPA), we measured the aversive learned behavior, which directly reflects the affective component of visceral pain [15,16]. When CRD was paired with a conspicuous environmental context, the rats spent significantly less time in this compartment on the post-conditioning test days than the pre-conditioning days. Our previous observation and current data showed no anxiogenic behavior, and no significant differences in locomotor behavior were observed during the conditioning days. Our previous publications described the experimental details [15,16]. Briefly, the apparatus consisted of three wooden compartments (45 × 45 cm each). Two compartments were conditioning compartments, with one or the other being paired with CRD and any other treatment, and the third was a neutral compartment. Each of the three compartments was characterized by distinct visual and olfactory cues. One conditioning compartment had horizontal stripes on the walls and an odor of 1.0% acetic acid, whereas the other had vertical stripes and a standardized cinnamon scent. Walls of a uniform color and no distinctive smell characterized the neutral compartment. Tactile coverings characterized the floors of the conditioning compartments. The neutral compartment had two doors, which opened to each conditioning compartment. During the conditioning, these openings could be closed to constrain the animal within a single conditioning compartment. The task comprised a pre-conditioning day (day 1), a conditioning phase (day 2–5) and post-conditioning days (a total of five test days). The animals were handled by the researcher for five minutes per day for the first two days before any behavioral testing. On day 1, the entrances connected to each compartment were opened, and the rat was allowed to move freely throughout the entire apparatus (i.e., all three compartments) for 20 min. The time spent by the rat in each compartment was recorded. No initial preferences for any compartments were detected on the pre-conditioning days, indicating that the rats did not prefer any one compartment to the others before conditioning. The conditioning phase comprised four consecutive days of being paired with the subthreshold pressure (<35 mm Hg) of CRD in each of the conditioning compartments (A and B), and then the rats were allowed to explore one of the conditioning compartments for 45 min freely or with another treatment (i.e optogenetic and Gi activation of ACC astrocytes). After the conditioning phase, each rat was allowed to move freely throughout the three compartments for 20 min without aversive stimulus (CRD) or any other treatment. The time spent in each compartment was recorded. The amount of time spent in the conditioning compartment (paired with CRD) on the post-conditioning test days was subtracted from the time spent in the same compartment on the pre-conditioning day. These processes produced a CPA score for each rat.

### 2.7. In Vivo Microdialysis

The details of the methods for the in vivo microdialysis are described in our previous publication [10]. Briefly, the rats were anesthetized (urethane; 1.6 g/kg, i/p), and a microdialysis guide cannula (CMA 11; CMA Inc., Stockholm, Sweden) was implanted unilaterally into the right ACC (AP = +2.8 mm, ML = ±0.8 mm and DV = 2.8 mm from the skull surface). The rats were handled for 10 min every day for five days prior to the microdialysis. Before starting the training, every day for four consecutive days, a microdialysis probe (CMA 11 elite, 2 mm membrane, CMA Inc., Stockholm, Sweden) was inserted into the guide cannula and perfused with artificial cerebrospinal fluid (aCSF) at a rate of 2 µL/min, and the lysate was collected every 10 min. The first two samples were discarded. After 20 min of baseline collection, the rats were trained in the conditioning chamber for 45 min paired with a pharmacological drug, optogenetic and chemogenetic manipulation of the ACC astrocytes. The sample collection was then continued for 20 min immediately after the conditioning training.

#### 2.7.1. Experiment 1: Measurement of the Training-Dependent Extracellular L-Lactate Level in the ACC

To test whether the CPA training led to extracellular L-lactate release in the ACC of the freely moving rats, we implanted a microdialysis probe into the ACC (AP = 2.8 mm, ML = 0.8 and DV = 2.5–3.0 mm). After recovery, the rats received CPA training for four consecutive days. On each conditioning training day, two dialysates were collected continuously for 20 min from each rat: one before the start of training, which served as the baseline, and the other immediately after the end of the training.

#### 2.7.2. Experiment 2: Extracellular L-Lactate Level in Response to Bilateral Infusion of DAB into the ACC

To see the effect of the exogenous administration of the glycogen phosphorylation inhibitor DAB (1 µL; 300 µM) on the extracellular L-lactate release from the ACC, we implanted a bilateral chronic cannula into the ACC, and a microdialysis probe was also implanted rostrally angled at 10^0^ toward the cannula. After one week of recovery, the rats received saline or DAB every day for four consecutive days, 15 min before the start of conditioning. Dialysates were continuously collected for 20 min immediately after the completion of the conditioning training.

#### 2.7.3. Experiment 3: L-Lactate Level in Response to Optogenetic Activation of ACC Astrocytes

To determine whether the optogenetic activation of ACC astrocytes modulated the extracellular L-lactate release in the ACC, an AAV8-gfaABC1D-hChR2(H134R)-eGFP (5.5 × 10^12^, 0.45 µL; Taitool Bioscience, Shanghai, China) was injected into the ACC (AP = 2.8 mm, ML = 0.8 mm and DV = 2.8 mm). After the viral injection, the rats received bilateral surgical implantation of the chronic fiber optic cannula into the ACC at 0.3 mm above the injection site to deliver the blue light to activate the ACC astrocytes. A microdialysis probe was also implanted rostrally angled at 10^0^ toward the fiberoptic cannula into the right ACC. The rats were allowed three weeks to express the virus. After 20 min of baseline collection on each training day, the rats received a local injection of DAB (1 µL; 300 µM) 15 min before the conditioning training. Then during conditioning, the ACC astrocytes were optically stimulated with a blue light pulse of 45 ms through an implanted optical fiber at a 20 Hz frequency (3 min ON; 3 min OFF) for 45 min of conditioning. The control group of rats only received DAB before each training day without optical stimulation of ACC astrocytes. Dialysates were continuously collected for 20 min immediately after the completion of the conditioning.

#### 2.7.4. Experiment 4: L-Lactate level in response to chemogenetic manipulation of ACC astrocytes

To investigate whether Gi pathway activation in ACC astrocytes affects the extracellular L-lactate release, an AAV5-GFAP-hM4D(Gi)-mCherry (5.5 × 10^12^, 0.45 µL; Addgene, Watertown, MA, USA; 50479-AAV5) was injected into the ACC (AP 2.8 mm, ML 0.8 mm and DV 2.8 mm). After the viral injection, the rats received a surgical implantation of a microdialysis probe into the right ACC. The rats were allowed three weeks to express the virus. After 20 min of baseline collection on each training day, the rats underwent conditioning. The Gi pathway (hM4Di receptors) of the ACC astrocytes was stimulated with a chemogenetic drug, CNO (1 mg/kg b.w.; i.p.), thirty minutes before the conditioning training. The control rats only received saline. Dialysates were continuously collected for 20 min after the completion of the conditioning.

#### 2.7.5. L-Lactate measurement and analysis

All dialytic samples were stored at −80 °C until the assays. According to the manufacturer’s instructions, the dialysate samples were analyzed using a Lactate Fluorescence Assay Kit (Abcam, Boston, MA, USA). In brief, 5–10 μL of the dialysate sample was diluted with lactate assay buffer to a final volume of 50 μL in each well. A total of 50 μL of the reaction mix (46 μL lactate assay buffer, 2 μL probe and 2 μL enzyme mix) was added into each well containing the lactate standard or the testing samples. The reaction was incubated for 30 min at room temperature, protected from light. The lactate concentration was measured in a microplate reader. The baseline lactate concentrations showed inter-individual differences [19]. The data from each animal are expressed as percentages, and the individual values were calculated accordingly for each animal. The microdialysis data were analyzed using a two-way repeated-measures ANOVA followed by post hoc Bonferroni multiple comparison tests, and the results are expressed as the mean ± SEM.

### 2.8. Immunohistochemistry

For the immunostaining procedures, the rats were anesthetized with urethane (1.6 g/kg, i/p) after completing the behavioral study and perfused with ice-cold PBS followed by 4% paraformaldehyde (PFA). Then brain samples were incubated in PFA overnight and transferred to a 30% sucrose solution for three days. The brains were cryosectioned (Lecia CM3050 S Cryostat) at a thickness of 40 μm (coordinates 3.8 to 2.2 mm from bregma). The sections were then blocked by 10% normal goat serum in PBS with 0.3% Triton X-100 for 2 h and incubated with primary antibodies to mouse anti-GFAP (1:500, Sigma-Aldrich, St. Louis, MO, USA; catalog no. G3893), rabbit anti-GFAP (1:500, Abcam, Boston, MA, USA; catalog no. ab7260), mouse anti-NeuN (1:1000; Millipore, Burlington, MA, USA; catalog no. MAB-377), and rabbit anti-S100β (1:500, Abcam, Boston, MA, USA; catalog no. ab41548). The sections were then washed and labeled with an appropriate Alexa fluor secondary antibody (1:500). Finally, the sections were mounted onto microscope slides and a fluorescent mounting medium (DAKO) and covered with coverslips. The sections were photographed using an LSM 880 Zeiss laser scanning microscope. To make the analysis comparable, the quantitative images between the comparative groups were acquired under the 40 × oil objective on the same day and with the same perimeters of the confocal microscope system setting.

### 2.9. Western Blot Analysis

The sample preparation was carried out as described in our previous publication [10]. Briefly, to measure the expression of learning-dependent genes, the ACC was rapidly dissected 90 min after the CPA training, alone or paired with a specific treatment (final day of conditioning) using an anodized aluminum brain slicer (Braintree Scientific Instruments, Braintree, MA, USA) followed by homogenization in buffer containing 10 mM HEPES, 1 mM EDTA, 2 mM EGTA, 0.5 mM DTT, phosphatase, and protease inhibitor cocktails (Sigma–Aldrich, St. Louis, MO, USA). The homogenates were then centrifuged at 10,000× *g* for 30 min at 4 °C. The supernatant was collected and considered as the total protein for the Western immunoblot analysis. The total protein content of each brain homogenate was determined using the Pierce^TM^ BCA protein assay kit (ThermoScientific, Rockford, IL, USA). Forty micrograms of whole protein extract/lane were resolved using 10 or 15% SDS-PAGE and analyzed by Western blot. The membranes were blocked in 5% non-fat milk in TBST buffer, and the following primary antibodies were diluted in block buffer: rabbit anti-pCREB (1:1000; Millipore, Burlington, MA, USA; catalog no. 06–519), rabbit anti-CREB (1:1000; Millipore, Burlington, MA, USA; catalog no. 04–767), and rabbit anti-ERK1/2 (1:1000; Cell Signaling Technology, Danvers, MA, USA; catalog no. 197G2) and incubated overnight at 4 °C. α-Tubulin (1:5000; Sigma–Aldrich, St. Louis, MO, USA; catalog no. T6074) or β actin (1:5000; ImmunoWay, Plano, TX, USA; catalog no. YM3028) was used as the loading normalizing control. The membranes were washed three times in TBST for 10 min each and then incubated with horseradish peroxidase-coupled specific secondary antibodies, goat anti-rabbit IgG and goat anti-mouse IgG, where appropriate (1:4000; Invitrogen, Waltham, MA, USA), in TBST for 1 h at room temperature. The western blots were visualized using the Immobilon^®^ Western chemiluminescent HRP substrate (EMD Millipore Corporation, Burlington, MA, USA, catalog no. WBKLS0500). The images were captured and processed by a gel documentation system (Azure Biosystems), and a quantitative densitometric analysis was conducted using NIH ImageJ software. The band intensities obtained after the densitometry were calculated as the ratios of the target antibody to tubulin or actin. For the quantification of the pCREB and ERk1/2, both bands were averaged. Moreover, pCREB was normalized with its nonphosphorylation form of CREB (e.g., pCREB/CREB).

### 2.10. Statistical Analysis

The data are presented as the mean ± SEM, and the statistical analysis, where appropriate, was carried out using GraphPad Prism 9.5.0 (730) (Graph Pad, San Diego, CA, USA). The behavioral and in vivo microdialysis data were analyzed by two-way repeated-measure ANOVA adjusted by the Bonferroni post hoc test. For the Western blot data, the Mann–Whitney U test was applied.

## 3. Results

### 3.1. Conditioning Training-Induced Lactate Release from ACC Astrocytes Contributes to Aversive Memory Formation

**Figure 1:**
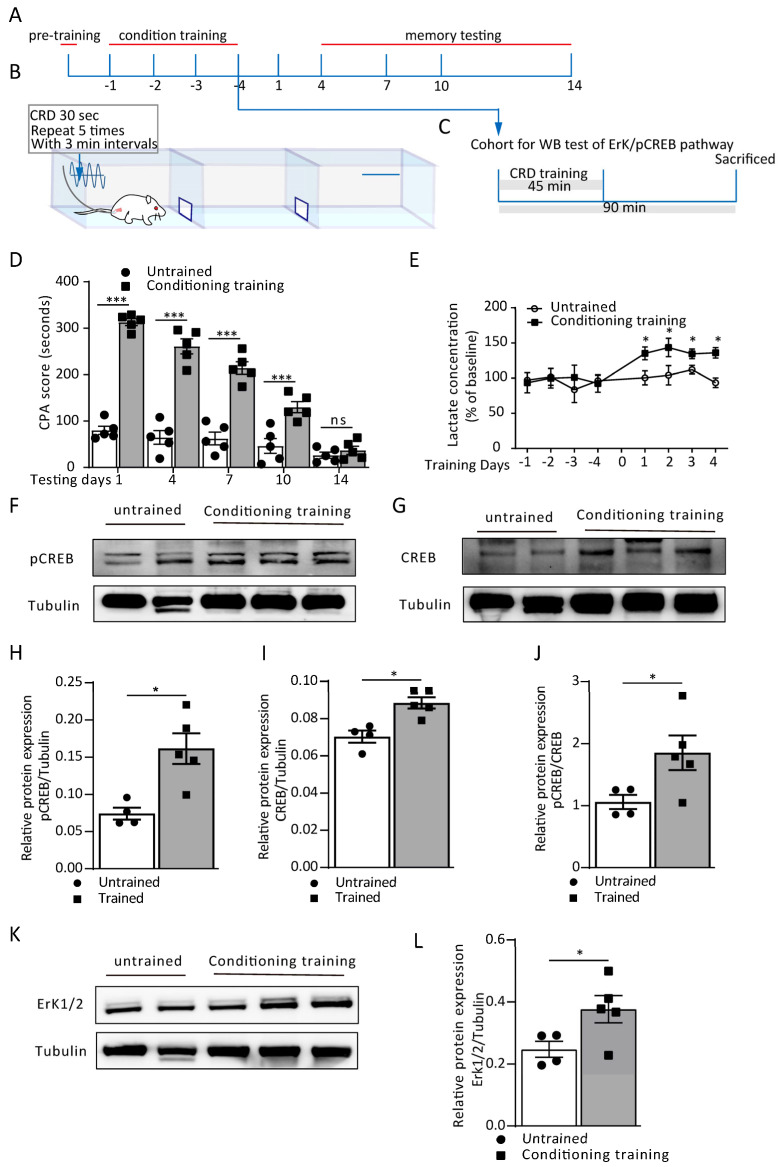
Conditioning training induced lactate release from ACC astrocytes during aversive memory formation. (**A**) Schematic protocol of conditioned place avoidance (CPA) and (**B**) Schematic of CPA behavioral paradigm. (**C**) The experimental timeline for the collection of Western blot samples for the plasticity-related proteins. (**D**) CPA score in the untrained and conditioning trained rats on testing day 1 to day 14 (untrained vs. conditioning training; day 1: 80.0 ± 8.95 vs. 313.2 ± 7.42, ^***^ *p* < 0.0001; day 4: 74.8 ± 14.62 vs. 261.0 ± 16.09, ^***^ *p* < 0.0001; day 7: 62.4 ± 13.62 vs. 214.40 ± 13.49, ^***^ *p* < 0.0001; day 10: 46.6 ± 15.95 vs. 130.0 ± 11.88, ^***^ *p* < 0.0001; day 14: 26.60 ± 6.60 vs. 37.0 ± 8.86, ^ns^ p = 0.9817; two-way ANOVA with Bonferroni post hoc test; *n* = 5 rats/group). Data are presented as the mean  ±  SEM. (**E**) ACC extracellular lactate concentration in the untrained and conditioning trained rats from training day 1 to day 4 (untrained vs. conditioning training; day 1: 100.47 ± 10.04 vs. 141.95 ± 5.88, ^*^ *p* = 0.0194; day 2: 104.03 ± 13.83 vs. 143.58 ± 12.93, ^*^ *p* = 0.0268; day 3: 100.49 ± 4.43 vs. 137.88 ± 5.38, ^*^ *p* = 0.0381; day 4: 93.47 ± 6.82 vs. 136.11 ± 7.48, ^*^ *p* = 0.016; two-way ANOVA with Bonferroni post hoc test). Data are presented as a % of the baseline ± SEM. (**F**–**L**) Representative images and Western blot analyfsis of the pCREB and pCREB/CREB (**F**,**H**,**J**; ^*^ *p* = 0.0317), CREB (**G**,**I**; ^*^ *p* = 0.0159) and Erk 1/2 (**K**,**L**; ^*^ *p* = 0.0435) from untrained and conditioning trained rats (Mann–Whitney test; *n* = 4 in the untrained group, *n* = 5 in the conditioning trained group). ns = no significance, *p* > 0.05.

During pain-aversive memory learning, astrocytes in the ACC modulate neuronal plasticity by L-lactate fed through the astrocyte–neuron lactate shuttle (ANLS). Here, we utilized a visceral pain model that combined colorectal distension (CRD) with a conditioned place avoidance (CPA) behavior paradigm (Figure 1B). The conditioning memory was tested on post-conditioning days 1, 4, 7, 10 and 14 to assess aversive memory retrieval (Figure 1A). Next, to determine the protein expression of the Erk/pCREB pathway, a cohort of the rats was sacrificed on day 4, 90 min after the conditioning training (Figure 1C). We found that the rats successfully established aversive memory after four days of CRD-paired conditioning training. From the CPA score recorded on the testing day, the conditioning training group showed a significant increase compared to the untrained group from day 1 to day 10 (treatment: F_(1, 40)_ = 305.2, *p* < 0.0001; time (days): F_(4, 40)_ = 56.12, *p* < 0.0001; interaction (treatment × time): F_(4, 40)_ = 26.67, *p* < 0.0001; Figure 1D). However, the aversive memory also indicated a trend of extinction, since there was no difference detected on day 14 between the two groups (*p* = 0.9817, Figure 1D). We then performed an in vivo microdialysis in the ACC and detected the L-lactate levels at separate time points on four consecutive CRD-paired conditioning training days to assess the role of ACC extracellular L-lactate in aversive memory establishment. The L-lactate level remained at the baseline in the rats without training, while conditioning training led to a substantial increase in the extracellular lactate concentration in the ACC. Compared to the untrained group, the normalized L-lactate concentration was significantly higher (Figure 1E).

The cyclic AMP (cAMP)-responsive element-binding protein (CREB) is one of the many transcription factors involved in memory formation and consolidation. To verify whether the conditioning training stimulated CREB phosphorylation, we performed a Western blot to test the p-CREB and CREB expression in the ACC. Compared with the untrained group, the pCREB/CREB ratio significantly increased in the trained group (*p* = 0.0480, Mann–Whitney test; Figure 1F,J), indicating the phosphorylation of CREB during the conditioning training. Meanwhile, since the growth factor/receptor tyrosine kinase activity Erk pathway is a classical upstream signaling cascade of CREB phosphorylation, we then tested the Erk1/2 protein expression. Identical to p-CREB/CREB, Erk1/2 showed a significant increase in the trained group (*p* = 0.0435, Mann–Whitney test; Figure 1K,L). Together, these data demonstrate that the CPA training facilitated aversive memory formation and consolidation, resulting in L-lactate release in the ACC and activation of the Erk/p-CREB pathway.

**Figure 2:**
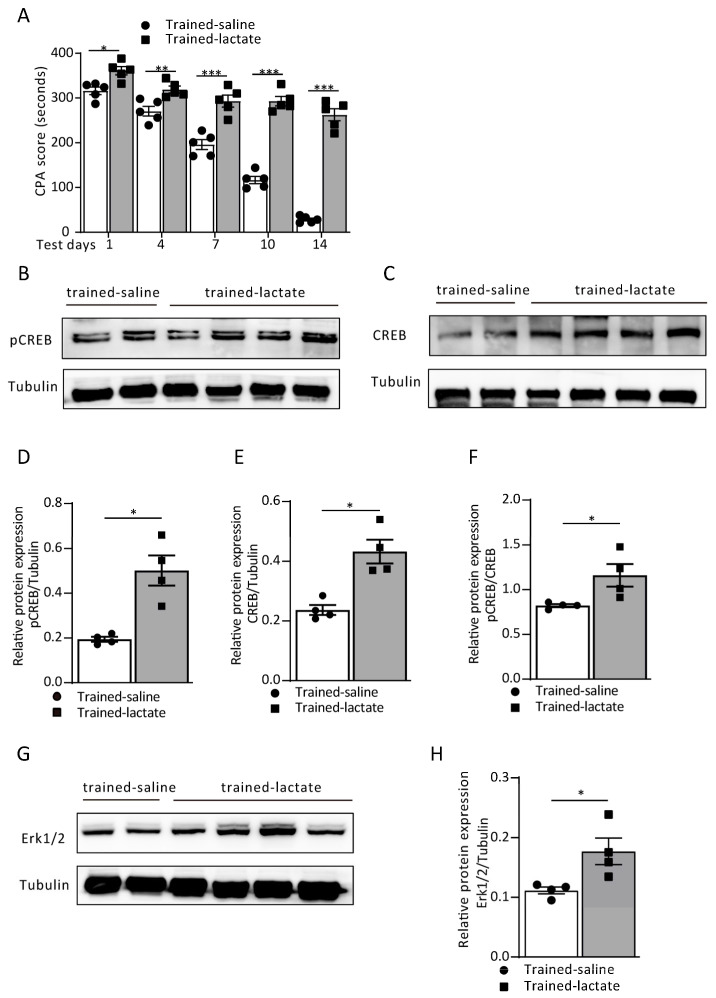
Exogenous local L-lactate reinforces aversive memory formation. (**A**) CPA score in the trained-saline and trained-lactate group recorded on test days 1, 4, 7, 10 and 14, respectively (trained saline vs. trained lactate; day 1: 316.0 ± 8.34 vs. 361.0 ± 9.14, ^*^ *p* = 0.0144; day 4: 270.60 ± 10.91 vs. 319.0 ± 7.69, ^**^ *p* = 0.0074; day 7: 196.0 ± 11.20 vs. 293.20 ± 13.27, ^***^ *p* < 0.0001; day 10: 116.80 ± 8.16 vs. 293.0 ± 10.48, ^***^ *p* < 0.0001; day 14: 28.40 ± 3.23 vs. 262.60 ± 13.58, ^***^ *p* < 0.0001; two-way ANOVA with Bonferroni post hoc test; *n* = 5 each group). Data are expressed as the mean ± SEM. (**B**–**H**) The representative Western blot images and analysis of the pCREB and pCREB/CREB (**B**,**D**,**F**; ^*^ *p* = 0.0286), CREB (**C**,**E**; ^*^ *p* = 0.0286) and Erk 1/2 (**G**,**H**; ^*^ *p* = 0.0286) from the trained-saline and trained-lactate groups (Mann–Whitney test; *n* = 4 each group).

Our previous research demonstrated that L-lactate facilitates rats’ decision making through the BLA-ACC neural network. We asked whether L-lactate reinforces aversive memory formation and consolidation. We performed an exogenous infusion of L-lactate on every training day, wherein saline or L-lactate (1 µL; 10 mM) was injected into each hemisphere of the ACC immediately before the conditioning training in the saline and L-lactate group of rats, respectively. We recorded the CPA score on test days 1, 4, 7, 10 and 14. From the CPA score on each test day, we found that the exogenous infusion of L-lactate noticeably reinforced the aversive memory recall and maintained it longer than the saline group (treatment: F_(1, 40)_ = 359.3, *p* < 0.0001; time (days): F_(4, 40)_ = 113.1, *p* < 0.0001; interaction (treatment × time): F_(4, 40)_ = 34.16, *p* < 0.0001; Figure 2A). More interestingly, infusion of L-lactate in the ACC before the training also induced Erk/p-CREB pathway activation (Figure 2B–H). The quantitative Western blot analysis of the L-lactate-infused rats revealed a significant increase in the ratio of pCREB/CREB expression (*p* = 0.0286, Mann–Whitney test; Figure 2F). Meanwhile, the expression of the upstream Erk1/2 pathway showed the same trend (*p* = 0.0286, Mann–Whitney test; Figure 2G,H), indicating that exogenous L-lactate infusion enhances aversive memory through the Erk/pCREB pathway.

### 3.2. Blocking Astrocytic Glycogenolysis in the ACC Disrupts Aversive Memory by Decreasing the L-Lactate Level

**Figure 3:**
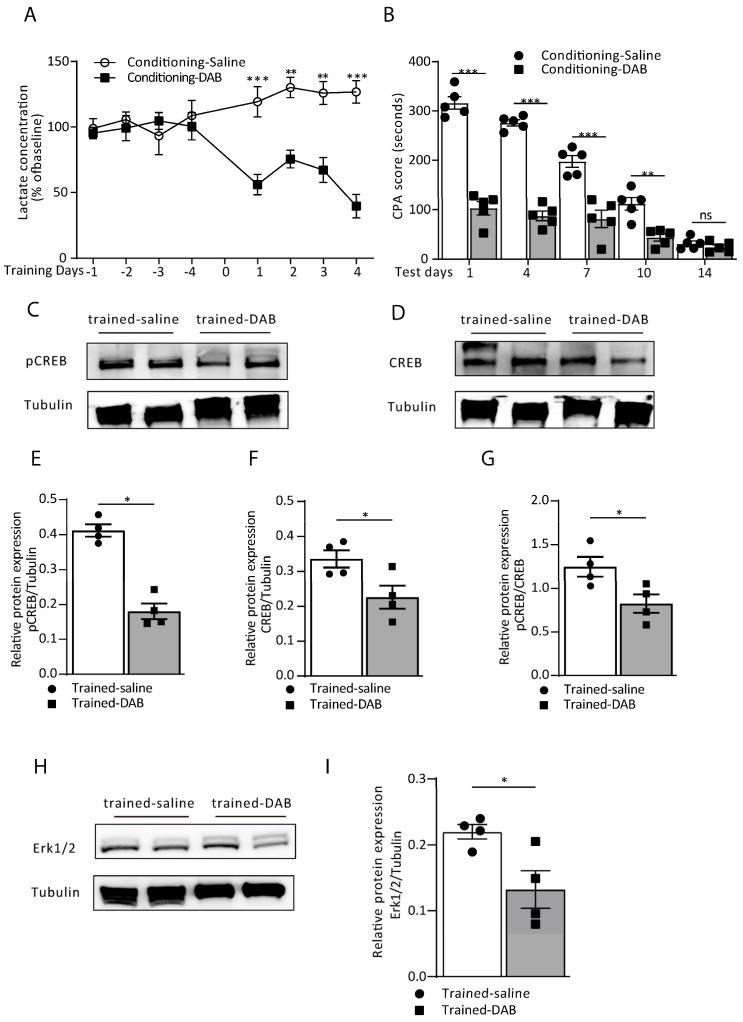
Blocking astrocytic glycogenolysis in the ACC disrupts aversive memory by decreasing the L-lactate level. (**A**) In vivo extracellular L-lactate levels from ACC lysate of the saline and DAB groups of rats from conditioning training day 1 to day 4 (saline vs. DAB; day 1: 109.25 ± 3.39 vs. 56.02 ± 3.34, ^***^ *p* = 0.0006; day 2: 116.82 ± 7.83 vs. 76.50 ± 6.84, ^**^ *p* = 0.0055; day 3: 112.58 ± 6.43 vs. 67.18 ± 9.50, ^**^ *p* = 0.0025; day 4: 116.82 ± 4.97 vs. 39.69 ± 8.87, ^***^ *p* < 0.0001; two-way ANOVA with Bonferroni post hoc test; *n* = 5 each group). Data are presented as the % of the baseline ± SEM. (**B**) CPA score in the saline and DAB groups of rats on testing day 1 to day 14 (saline vs. DAB; day 1: 316.40 ± 12.67 vs. 103.0 ± 13.36, ^***^ *p* < 0.0001; day 4: 267.0 ± 6.14 vs. 87.40 ± 9.61, ^***^ *p* < 0.0001; day 7: 196.80 ± 11.90 vs. 81.40 ± 16.31, ^***^ *p* < 0.0001; day 10: 112.0 ± 12.82 vs. 44.0 ± 7.47, ^**^ *p* = 0.004; day 14: 31.60 ± 5.22 vs. 24.40 ± 4.69, ^ns^ p = 0.9941; two-way ANOVA with Bonferroni post hoc test; *n* = 5 each group). Data are expressed as the mean ± SEM. (**C**–**I**) The representative Western blot images and analysis of the pCREB and pCREB/CREB (**C**,**E**,**G**; ^*^ *p* = 0.0386), CREB (**D**,**F**; ^*^ *p* = 0.0143) and Erk 1/2 (**H**,**I**; ^*^ *p* = 0.0450) from the saline and DAB groups of rats (Mann–Whitney test; *n* = 4 each group). ns = no significance, *p* > 0.05.

1,4-Dideoxy-1,4-imino-D-arabinitol (DAB) does not affect the glucose breakdown but inhibits glycogen phosphorylase, thus reducing the lactate level by blocking the glycogenolysis in astrocytes and thereby interrupting memory consolidation. We next infused DAB on every training day to detect the extracellular L-lactate levels in the ACC and then tested its effect on memory formation. The exogenous infusion of DAB (1 µL; 300 µM) 15 min before the conditioning training found that the extracellular L-lactate level, collected by microdialysis after the training, was significantly decreased compared to the saline group (Figure 3A). In addition, the DAB group also showed disruption of conditioning memory; on test day 1 to day 14. The CPA score of the DAB group dropped considerably compared to the saline group (treatment: F_(1, 40)_ = 298.4, *p* < 0.0001; time (days): F_(4, 40)_ = 93.97, *p* < 0.0001; interaction (treatment x time): F_(4, 40)_ = 30.56, *p* < 0.0001; Figure 3B), indicating that the DAB group failed to recall aversive memory. Identically, the inhibition of astrocytic glycogenolysis reduces plasticity-related protein expression in ACC neurons. After training, we tested the Erk/pCREB pathway protein expression (Figure 3C–I) and found that the pCREB/CREB ratio was lower than the saline group (*p* = 0.0386, Mann–Whitney test; Figure 3G). Similarly, the upstream Erk1/2 expression was also reduced by the DAB infusion (*p* = 0.0450, Mann–Whitney test; Figure 3H,I). These results indicate that the exogenous infusion of DAB in the ACC disrupts neuronal plasticity-related protein expression, impacting aversive memory through the reduced L-lactate production in astrocytes.

**Figure 4:**
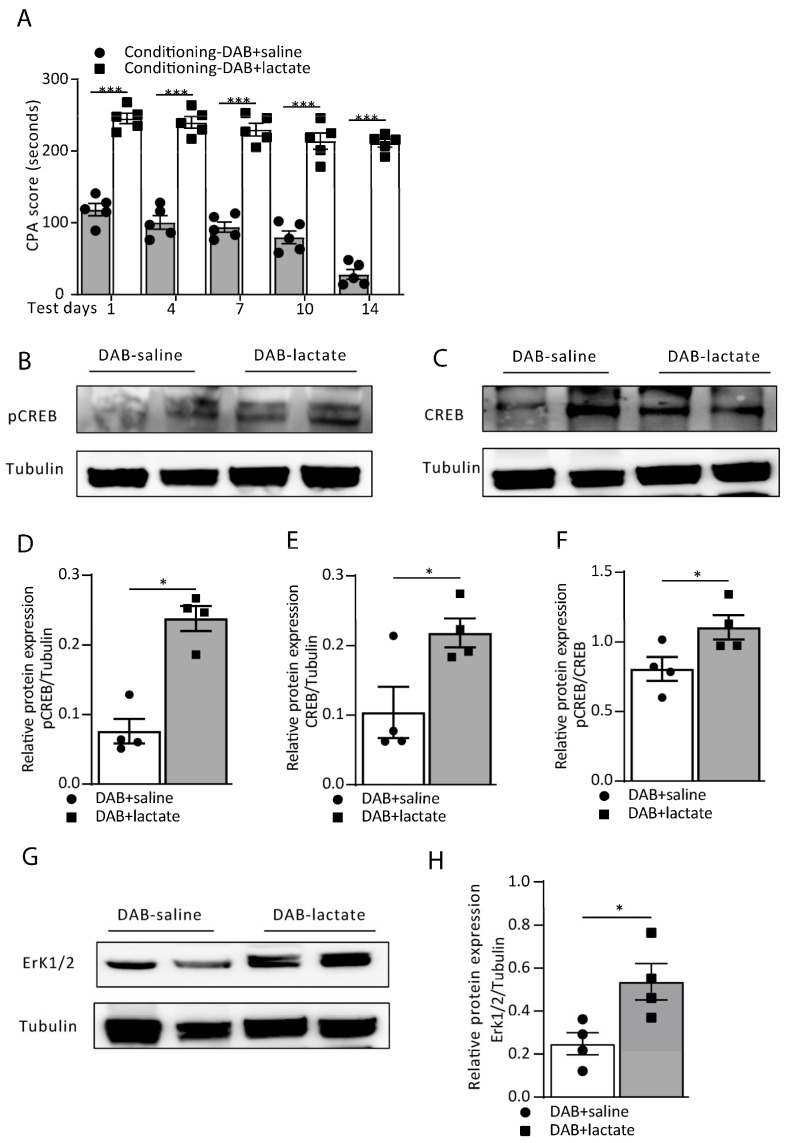
Exogenous L-lactate rescues DAB-induced aversive memory impairment. (**A**) The CPA score from the DAB+saline and DAB+lactate groups of rats on testing day 1 to day 14 (DAB-saline vs. DAB-lactate; day 1: 118.40 ± 8.60 vs. 245.60 ± 7.18, ^***^ *p* < 0.0001; day 4: 100.6 ± 9.53 vs. 240.0 ± 8.08, ^***^ *p* < 0.0001; day 7: 94.0 ± 7.13 vs. 230.0 ± 8.77, ^***^ *p* < 0.0001; day 10: 79.8 ± 8.90 vs. 213.8 ± 11.47, ^***^ *p* < 0.0001; day 14: 28.20 ± 6.92 vs. 211.20 ± 5.50, ^***^ *p* < 0.0001; two-way ANOVA with Bonferroni post hoc test; *n* = 5 each group). Data are expressed as the mean ± SEM. (**B**–**H**) The representative Western blot images and analysis of the pCREB and pCREB/CREB (**B**,**D**,**F**; ^*^ *p* = 0.0286), CREB (**C**,**E**; ^*^ *p* = 0.0243) and Erk 1/2 (**G**,**H**; ^*^ *p* = 0.0286) from the DAB-saline and DAB-lactate groups of rats (Mann–Whitney test; *n* = 4 each group).

Since DAB effectively reduced the extracellular L-lactate levels in the ACC and consequently disrupted aversive memory retrieval by Erk/pCREB pathway inhibition, can exogenous infusion of L-lactate rescue the DAB-induced amnesia? To test this, we injected exogenous L-lactate (10 mM) together with DAB (300 µM) fifteen minutes before the conditioning training in the DAB+lactate group and DAB+saline group. We tested for memory recall on the test days and found that L-lactate successfully rescued the DAB-induced amnesia compared to the DAB+saline group (treatment: F_(1, 40)_ = 740.8, *p* < 0.0001; time (days): F_(4, 40)_ = 16.58, *p* < 0.0001; interaction (treatment × time): F_(4, 40)_ = 3.56, *p* = 0.0142; Figure 4A). Moreover, the expression of the plasticity-related proteins of the Erk/pCREB pathway in ACC neurons also showed improvement (Figure 4B–H). The expression of the pCREB/CREB ratio was enhanced by adding L-lactate (*p* = 0.0286, Mann–Whitney test; Figure 4F), and similar effects were also noticed in the Erk1/2 expression (*p* = 0.0286, Mann–Whitney test; Figure 4G,H). Together these data demonstrate that exogenous lactate administration upregulated the learning-dependent plasticity changes and rescued the DAB-induced aversive memory formation.

### 3.3. Optogenetic Activation of ACC Astrocytes Reverses Aversive Memory Formation from Glycogenolysis Blocking by Increasing the L-Lactate Level

**Figure 5:**
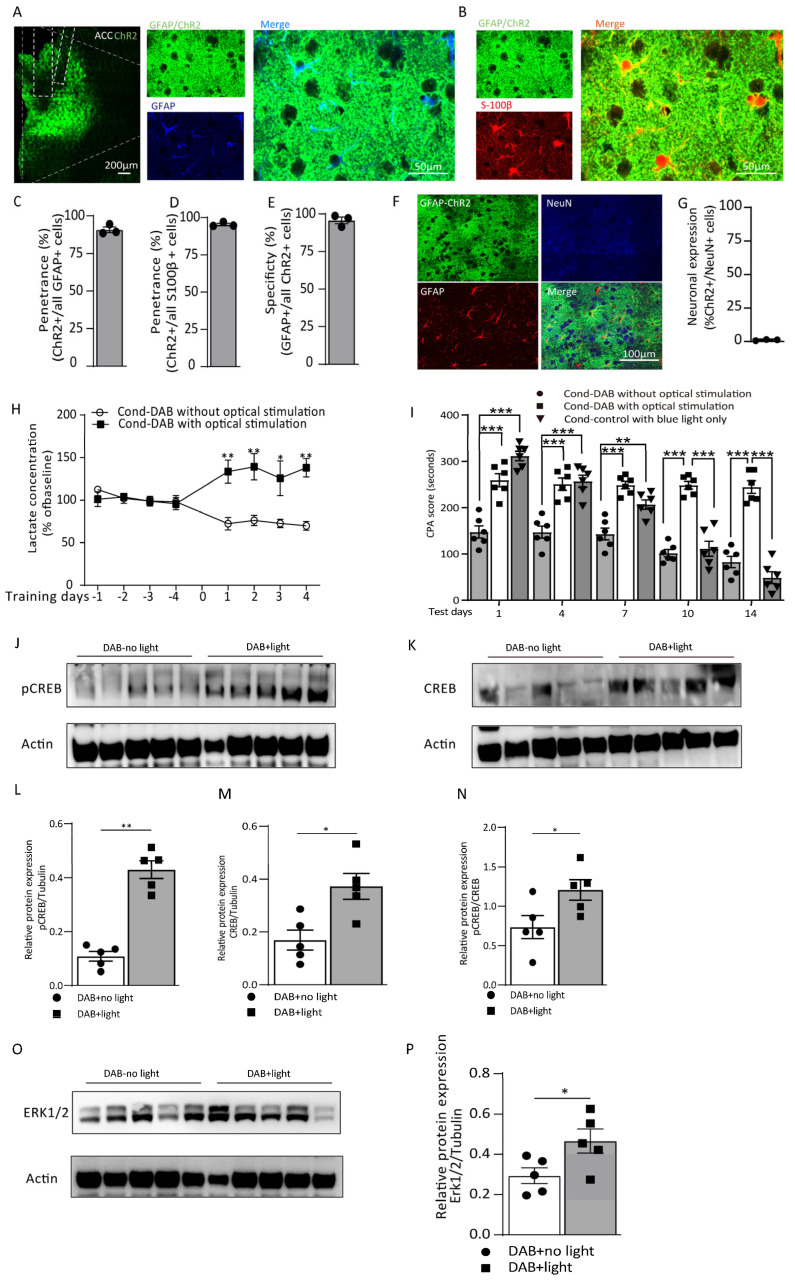
Astrocytic optogenetic activation in the ACC reverses the aversive memory formation from glycogenolysis blocking by increasing the L-lactate level. (**A**) Left: representative image from rat expressing AAV8-gfaABC1D-hChR2(H134R)-eGFP in ACC astrocytes (scale bar: 100 µm). Right: representative fluorescent images of GFAP colocalized with ChR2 (scale bar: 50 µm). (**B**) Representative fluorescent images of S-100β colocalized with ChR2 (scale bar: 50 µm). (**C**,**D**) Penetrance of ChR2+ cells in the GFAP+ (90.74 ± 1.86% in C) and S100β+ cells (95.39 ± 0.83% in D). (**E**) Specificity of GFAP+ cells with ChR2+ (95.72 ± 2.18%). (**F**) Representative images of AAV8-gfaABC1D-hChR2(H134R)-eGFP costained with GFAP (red) and NeuN (blue). (**G**) % expression of AAV8-gfaABC1D-hChR2(H134R)-eGFP in the ACC neurons. (**H**) The extracellular L-lactate concentrations measured from ACC lysate in the DAB with optical stimulation and DAB without optical stimulation groups of rats (DAB-no light vs. DAB-light; day 1: 72.35 ± 7.38 vs. 133.57 ± 13.54, ^**^ *p* = 0.0079; day 2: 73.27 ± 6.10 vs. 139.30 ± 15.54, ^**^ *p* = 0.0063; day 3: 72.74 ± 4.91 vs. 125.76 ± 20.34, ^*^ *p* = 0.0225; day 4: 69.98 ± 5.29 vs. 138.02 ± 10.84, ^**^ *p* = 0.0033; two-way ANOVA with Bonferroni post hoc test; *n* = 5 each group). Data are presented as the % of the baseline ± SEM. (**I**) The CPA score was measured on testing day 1 to day 14 in the DAB-without optical stimulation, DAB-optical stimulation and control-with blue light groups of rats (DAB-no light vs. DAB-light; day 1: 147.5 ± 13.04 vs. 259.33 ± 13.91, ^***^ *p* < 0.0001; day 4: 147.16 ± 12.80 vs. 250.83 ± 12.96, ^***^ *p* < 0.0001; day 7: 143.16 ± 12.67 vs. 249.0 ± 8.03, ^***^ *p* < 0.0001; day 10: 101.66 ± 7.91 vs. 248.50 ± 8.21, ^***^ *p* < 0.0001; day 14: 28.66 ± 12.06 vs. 244.50 ± 13.37, *p* < 0.0001; two-way ANOVA with Bonferroni post hoc test; *n* = 6 each group). Data are expressed as the mean ± SEM. (**J**–**P**) The representative Western blot images and analysis of the pCREB and pCREB/CREB (**J**,**L**,**N**; ^**^ *p* = 0.0079, ^*^ *p* = 0.0456), CREB (**K**,**M**; ^*^p = 0.0159) and Erk 1/2 (**O**,**P**; ^*^ *p* = 0.0460) in the DAB-optical stimulation and DAB-without optical stimulation groups of rats (Mann–Whitney test; *n* = 5 in the DAB-optical stimulation group, *n* = 5 in the DAB-without optical stimulation group).

Optogenetic and chemogenetic tools allow for the real-time reversible manipulation of astrocytes in tandem with behavioral measurements. Firstly, we used an adeno-associated virus (AAV) construct with a GFAP promotor containing ChR2-EYFP and bilaterally injected it into the ACC to stimulate the ACC astrocytes. The expression of the vectors had a high penetrance and high specificity (Figure 5A–E). The costaining with a neuronal marker (NeuN) showed a 1% overlap with the GFAP-ChR2-EYFP (Figure 5F,G). On the conditioning training days, after 300 µM DAB was locally infused into the ACC, the astrocytes received optical stimulation with a blue light during conditioning, while no light stimuli were given to the control group. When the conditioning training was completed, extracellular dialysates were continuously collected for 20 min. Using the in vivo microdialysis, we found that the optogenetic stimulation of the astrocytes reversed the L-lactate decrease caused by the DAB glycogenolysis inhibition. The L-lactate level showed a slight increase from baseline, significantly higher than the DAB group without the blue light stimuli (Figure 5H). Similarly, the aversive memory was also rescued by the optogenetic activation of the astrocytes. On the test days, the group of rats that received the DAB infusion plus the optogenetic light stimulation during the training had a considerably higher CPA score than the DAB infusion rats without the optical stimuli (treatment: F_(2, 75_) = 137.2, *p* < 0.0001; time (days): F_(4, 75)_ = 45.60, *p* < 0.0001; interaction (treatment × time): F_(8, 75)_ = 19.88, *p* < 0.0001; Figure 5I). To further verify this result, we tested the protein expression of the Erk/pCREB pathway when the rats were sacrificed after the training on day 4 (Figure 5J–P). The pCREB/CREB ratio was significantly higher in the DAB-light group than in the DAB-no light group (*p* = 0.0317, Mann–Whitney test; Figure 5N).

### 3.4. Astrocytic Gi Pathway Activation in the ACC Disrupts Aversive Memory Formation by Decreasing the L-Lactate Level

**Figure 6:**
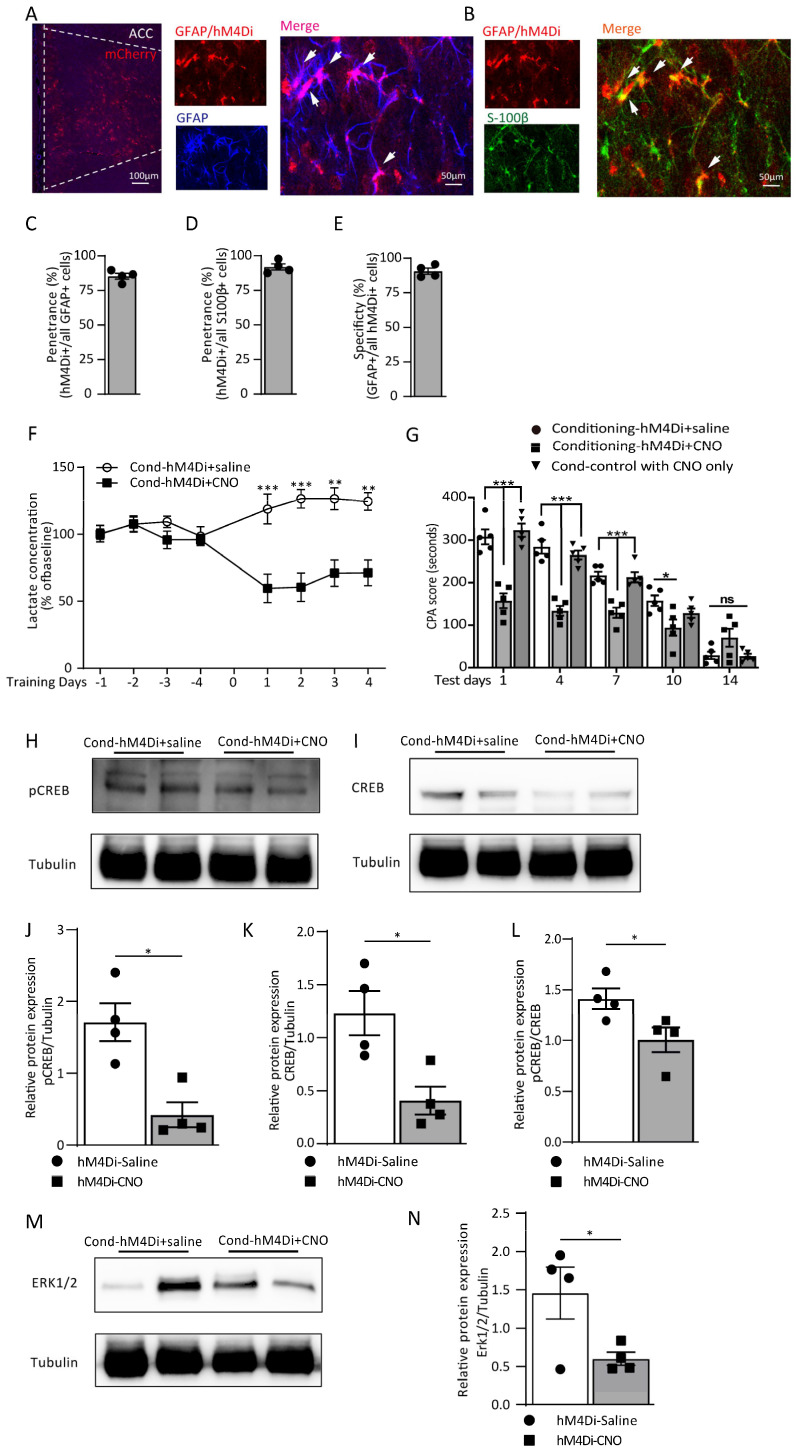
Astrocytic Gi pathway activation in the ACC disrupts aversive memory formation by decreasing the L-lactate level. (**A**) Left: representative image from rat expressing AAV5-GFAP-hM4Di-mCherry in ACC astrocytes (scale bar: 100 µm). Right: representative fluorescent images of GFAP colocalized with hM4Di-mCherrry in ACC astrocytes (scale bar: 50 µm). (**B**) Representative fluorescent images of S-100β colocalized with hM4Di-mCherrry in ACC astrocytes. (**C**,**D**) Penetrance of ChR2+ cells with GFAP+ (85.47 ± 2.12% in **C**) and S100β+ cells (92.10 ± 2.20% in **D**). (**E**) Specificity (90.66 ± 2.14%) of GFAP+ cells with ChR2+. (**F**) The extracellular L-lactate concentrations in the hM4Di-saline and hM4Di-CNO groups of rats in the conditioning training from day 1 to day 4 (hM4Di-saline vs. hM4Di-CNO; day 1: 118.80 ± 11.18 vs. 52.07 ± 10.74, ^***^ *p* = 0.0005; day 2: 126.48 ± 6.22 vs. 60.43 ± 10.68, ^***^ *p* = 0.0005; day 3: 126.45 ± 8.18 vs. 70.88 ± 9.96, ^**^ *p* = 0.0028; day 4: 124.43 ± 6.49 vs. 71.20 ± 9.68, ^**^ *p* = 0.004; two-way ANOVA with Bonferroni post hoc test; *n* = 4 each group). Data are presented as the % of the baseline ± SEM. (**G**) CPA score in the hM4Di-saline, hM4Di-CNO and control-CNO groups of rats on test day 1 to day 14 (hM4Di-saline vs. hM4Di-CNO; day1: 308.0 ± 17.35 vs. 157.0 ± 17.30, ^***^ *p* < 0.0001; day 4: 284.20 ± 15.95 vs. 133.60 ± 11.28, ^***^ *p* < 0.0001; day 7: 216.80 ± 9.17 vs. 129.0 ± 11.93, ^***^ *p* < 0.0001; day 10: 157.60 ± 12.42 vs. 94.44 ± 18.77, ^*^ *p* = 0.022; day 14: 29.20 ± 8.72 vs. 70.40 ± 21.17, *p* = 0.2933; two-way ANOVA with Bonferroni post hoc test; *n* = 5 each group). Data are expressed as the mean ± SEM. (**H**–**N**) The representative images and Western blot analysis of the pCREB and pCREB/CREB (**H**,**J**,**L**; ^*^ *p* = 0.0286, *p* = 0.0471), CREB (**I**,**K**; ^*^ *p* = 0.0470) and Erk 1/2 (**M**,**N**; ^*^ *p* = 0.0480) from the hM4Di-saline and hM4Di-CNO groups of rats (Mann–Whitney test; *n* = 4 each group). ns = no significance, *p* > 0.05.

As a tool for the chemogenetic manipulation of astrocytes, designer receptors are exclusively activated by designer drugs (DREADDs), which are used to activate the astrocytes’ Gi pathway. They have been confirmed to impair memory encoding and recall. Here, we employed an AAV5 carrying hM4Di fused to mCherry under the control of the astrocytic GFAP promoter. Vectors of AAV5-GFAP-hM4Di-mCherry were injected bilaterally into the ACC. CNO (1 mg/kg) i.p. was administered 30 min before the conditioning training in the CNO group, while saline was administered to the control group. Once the training was finished, the extracellular lysate was continuously collected for 20 min. The expression of the vectors of AAV5-GFAP-hM4Di-mCherry had a high penetrance and specificity (Figure 6A–E). The microdialysis showed that the Gi pathway activation was able to decrease the level of extracellular L-lactate, and the group of hM4Di-CNO expressed a decreasing trend than at baseline, and the discrepancy with the hM4Di-saline group was considerably high (Figure 6F). In addition, the retrieval of aversive memory on the testing day was dramatically disrupted, and the CPA score in the hM4Di-CNO group was significantly lower than for the hM4Di-saline group on testing day 1 to day 10 (treatment: F_(2, 60)_ = 53.55, *p* < 0.0001; time (days): F_(4, 60)_ = 118.3, *p* < 0.0001; interaction (treatment × time): F_(8, 60)_ = 11.56, *p* < 0.0001; Figure 6G), indicating that the Gi pathway activation during the training blocked the formation of aversive memory. To further assess this, the detection of the Erk/pCREB pathway protein expression was also conducted (Figure 6H–N). We found that both the pCREB/CREB and Erk expressions showed a dramatic reduction in the hM4Di-CNO group (*p* = 0.0471, *p* = 0.0480, Mann–Whitney test; Figure 6L,N).

**Figure 7:**
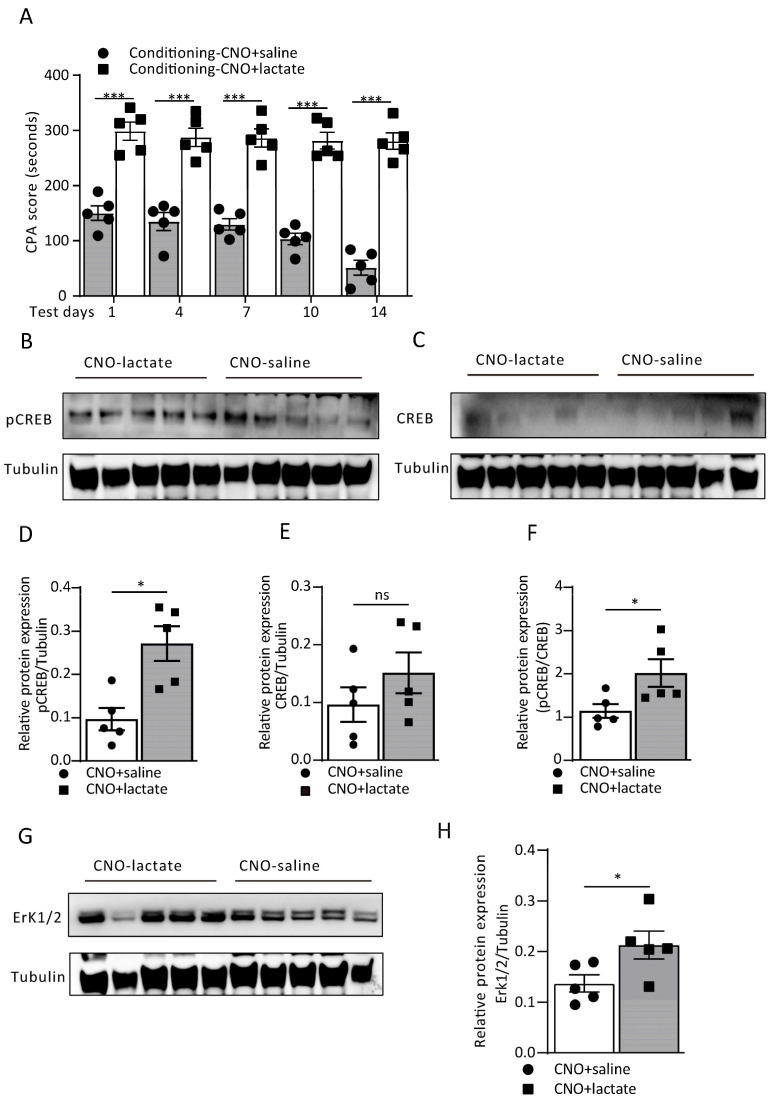
L-lactate infusion reverses aversion memory disrupted by ACC astrocytic Gi pathway activation. (**A**) CPA score recorded in CNO + saline and CNO + L-lactate groups of rats on test days 1, 4, 7, 10 and 14 (CNO-saline vs. CNO-lactate; day 1: 149.80 ± 13.21 vs. 298.60 ± 16.67, ^***^ *p* < 0.0001; day 4:134.80 ± 16.43 vs. 287.40 ± 16.69, ^***^p < 0.0001; day 7:129.60 ± 10.18 vs. 286.2 ± 16.33, ^***^p < 0.0001; day 10: 103.20 ± 10.30 vs. 281.40 ± 15.08, ^***^ *p* < 0.0001; day 14: 51.40 ± 13.52 vs. 280.60 ± 14.85, ^***^ *p* < 0.0001; two-way ANOVA with Bonferroni post hoc test; *n* = 5 each group). Data are expressed as the mean ± SEM. (**B**–**H**) The representative Western blot images and analysis of the pCREB and pCREB/CREB (**B**,**D**,**F**; ^*^ *p* = 0.0456), CREB (**C**,**E**; ^ns^ *p* = 0.156) and Erk ½ (**G**,**H**; ^*^ *p* = 0.0317) from the CNO + saline and CNO + L-lactate groups of rats (Mann–Whitney test; *n* = 5 for each group). ns = no significance, *p* > 0.05.

The above results demonstrate that astrocytic Gi pathway activation would decrease extracellular L-lactate levels. It is worth exploring whether the exogenous infusion of L-lactate would reverse either aversive memory or Erk/pCREB pathway protein expression. Therefore, we divided the rats into two groups, viz CNO i.p. administration plus L-lactate 10mM local injection into the ACC immediately before the conditioning training and saline i.p. instead. The result showed that the exogenous administration of 10 mM L-lactate reversed aversive memory during memory recall. The CPA score was significantly higher than in the CNO+saline group on testing days from day 1 to day 14 (treatment: F_(1, 40)_ = 355.0, *p* < 0.0001; time (days): F_(4, 40)_ = 4.71, *p* = 0.0033; interaction (treatment × time): F_(4, 40)_ = 2.64, *p* = 0.0477; Figure 7A). Identically, the administration of L-lactate in the Gi pathway activation group also improved the protein expression in the pCREB/Erk pathway (Figure 7B–H), as evidenced by a higher expression of both pCREB/CREB and Erk compared to the CNO-saline group (*p* = 0.0456, *p* = 0.0317, Mann–Whitney test; Figure 7F–H).

## 4. Discussion

Chronic pain is experienced when pain persists beyond the normal healing period. Patients’ psychological states, which are affected by cognitive and emotional factors, such as attention, an optimistic or pessimistic view, and empathic behavior, can control pain perception [13]. In recent years, numerous studies have established that pain results from the interactions between memory, attentional, cognitive and affective brain circuitry [1,6,9,13,27]. Cumulative evidence indicates that prolonged negative affection exists in the chronic pain state. The “secondary pain affect” includes enhanced aversive learning as well as emotional feelings regarding the long-term experience of pain (e.g., suffering) [28], which may be remembered or imagined [1,13]. Although the cognitive and emotional control of pain has been well documented in human subjects, animal experiments involving nociceptive behavioral responses in pain sensation and affection pose a significant challenge. Studies conducted on human and animal models have identified the anterior cingulate cortex (ACC) and its related structures as key areas in processing pain perception [2,3,4]. In the VH rat model, visceral allodynia and hyperalgesia are thought to be mediated by enhanced glutamate N-methyl-D-aspartate (NMDA)-receptor 2B activities [5,6,7,8,29]. We performed studies combining CRD with a conditioned place avoidance (CPA) paradigm in freely behaving rats [15,16]. We demonstrated that when CRD (≤35 mmHg, a subthreshold for pain perception) was paired with a distinct environmental context in the experimental apparatus, the rats spent significantly less time in this compartment on the post-conditioning test days compared with the pre-conditioning day, a result consistent with our previous reports [15,16]. These results suggested that the nociceptive stimulus given during the CRD-CPA tests resulted in a negative affective state. We and other investigators have shown that the ACC is essential for the aversive nature of nociceptor stimulation [15,16,27].

Astrocytes, the most abundant glial cells, respond to a variety of CNS insults through the process of reactive astrogliosis [30]. Astrocytes are able to differentiate the activity of discrete synapses that originate from different afferents, modify synaptic activity at surrounding synapses by releasing active substances to modulate the various neuronal circuits [17,18], and affect behavioral responses [26,31]. We previously showed that the optogenetic activation of ACC astrocytes facilitates decision making and rescued impaired decision making in rats with chronic visceral pain associated with increased ACC neuronal spike-theta synchrony [10]. Given that the astrocytes play an important role in regulating pain sensation and behaviors, here, we examined the role of astrocytes in visceral aversive behavior. To clarify the role of the astrocytic Gi pathway during aversive memory acquisition, we applied the DREADD method to manipulate ACC astrocytes, and the expressed Gi-coupled receptor hM4Di and virus expression in ACC astrocytes was confirmed. We discovered that astrocytic Gi pathway activation during learning and consolidation resulted in a specific impairment of pain aversive memory and that the same manipulation did not affect the pain sensation measured by CRD-induced visceral acute pain responses [7,8]. The mechanism underlying the astrocyte modulation of aversive memory is an intriguing open question. Glucose is the main source of carbohydrates for the brain. Approximately 85% of brain energy expenditure takes place within neurons [32]. Studies have demonstrated that glucose is not the preferred energy substrate during periods of intense neuronal activity, such as during long-term potentiation (LTP), when synaptic plasticity and synchronized brain network activity requires additional energy support [10,19,33].

It is plausible that in visceral pain-related conditioned place avoidance training and memory retrieval, the increased brain activity may lead to glutamate release at excitatory synapses, which enters astrocytes in a Na^+^-dependent manner leading to the activation of the Na^+^/K^+^-ATPase pump, which promotes the consumption of glucose and glycolysis and the production of L-lactate (astrocyte neuron lactate shuttle (ANLS) [21]. In this study, we performed an in vivo microdialysis and a L-lactate analysis and found that the CPA training caused marked increases in the L-lactate concentration in the ACC. Moreover, we found that infusion of L-lactate into the ACC during the training enhanced aversive memory recall. These observations suggest that astrocyte release of L-lactate may be involved in the modulation of visceral aversive learning and memory.

Astrocytes are the main sites of glycogen storage in the CNS [34]. Astrocytes can rapidly metabolize glycogen to lactate [35] and transport it to neurons during periods of neuronal activity. Next, we investigated whether astrocytic glycogen degradation [36] may lead to a surge in the local extracellular ACC lactate levels [22], which can then be provided to the neurons. DAB is an inhibitor of glycogen phosphorylase. Our previous study showed that infusion of DAB into the ACC impaired rat decision making accompanied with decreases in the lactate level in the physiological state [10]. Here, we showed that blocking lactate production with DAB impaired CPA training-induced increments of the extracellular lactate concentration in the ACC and blocked aversive memory formation. Next, we showed that the optogenetic activation of ACC astrocytes induced an increase in the L-lactate concentration and facilitated pain-related aversive behavior following the DAB infusion. These observations suggest that the additional energy demand induced by astrocyte glycogen degradation contributes to local lactate production. Moreover, lactate can be transported from astrocytes to the extracellular space thereafter entering neurons through monocarboxylate transporters (MCTs) [37,38]. Previously, we demonstrated that the MCT2 blocker 4-CIN prevented the effects of exogenous L-lactate infusion on decision-making behavior and rescued ACC synaptic long-term potentiation in visceral hypersensitivity rats [10]. Another study reported that the knockdown of MCT2 impairs memory, and both lactate and glucose failed to rescue this amnesia [19]. Lactate is imported from astrocytes to neurons wherein it is further metabolized in the mitochondria. Alternatively, the presence of a lactate-sensitive receptor in the brain [39] indicates that, apart from its metabolic function, lactate possesses a signaling function [38]. The GPCR protein is widely expressed in various types of neurons and presynaptic terminals and postsynaptic density, and it modulates neuronal activity in rodent and human brain tissue [40,41]. However, because current commercial HCAR1 antibodies lack specificity [42,43], the precise expression and localization of HCAR1 in the brain is still ambiguous.

In this study, we found that ACC astrocytic Gi activation almost completely disrupted the expression of pain aversive learning and the Erk/pCREB pathway. Note that the local infusion of L-lactate into the ACC restored this pathway protein’s expression following the CPA training. Consistent with what we and others have observed [10,44], L-lactate increased the expression of plasticity-related genes, including pCREB, Erk, c-Fos, Zif268 and BDNF, in cortical neurons. Therefore, L-lactate surely plays a role as a signaling molecule for neuronal plasticity [45].

## 5. Conclusions

In conclusion, we demonstrated that the L-lactate signaling between astrocytes and neurons in the ACC is necessary for visceral pain aversive learning and is essential for memory-related long-term synaptic changes in aversive memory formation. These results may also have important implications for understanding the mechanism of cognitive deficits in aversive memory and in chronic pain states in general.

## Data Availability

The data are available upon request from the corresponding author.

## References

[1] Baliki M.N., Apkarian A.V. (2015). Nociception, Pain, Negative Moods, and Behavior Selection. Neuron.

[2] Vogt B.A., Sikes R.W., Vogt L.J. (1993). Anterior Cingulate Cortex and the Medial Pain System. Neurobiology of Cingulate Cortex and Limbic Thalamus.

[3] Li Y. (2018). Synaptic Plasticity and Synchrony in the Anterior Cingulate Cortex Circuitry: A Neural Network Approach to Causality of Chronic Visceral Pain and Associated Cognitive Deficits. Adv Neurobiol..

[4] May A. (2008). Chronic pain may change the structure of the brain. Pain.

[5] Gao J., Wu X., Owyang C., Li Y. (2006). Enhanced responses of the anterior cingulate cortex neurones to colonic distension in viscerally hypersensitive rats. J. Physiol..

[6] Wang J., Zhang X., Cao B., Liu J., Li Y. (2015). Facilitation of synaptic transmission in the anterior cingulate cortex in viscerally hypersensitive rats. Cereb. Cortex.

[7] Cao Z., Wu X., Chen S., Fan J., Zhang R., Owyang C., Ying L. (2008). Anterior cingulate cortex modulates visceral pain as measured by visceromotor responses in viscerally hypersensitive rats. Gastroenterology.

[8] Fan J., Wu X., Cao Z., Chen S., Owyang C., Li Y. (2009). Up-regulation of anterior cingulate cortex NR2B receptors contributes to visceral pain responses in rats. Gastroenterology.

[9] Cao B., Wang J., Mu L., Poon D.C.-H., Li Y. (2016). Impairment of decision making associated with disruption of phase-locking in the anterior cingulate cortex in viscerally hypersensitive rats. Exp. Neurol..

[10] Wang J., Tu J., Cao B., Mu L., Yang X., Cong M., Aruna S.R., Rosa H.M.C., Liping W., Ying L. (2017). Astrocytic l-Lactate Signaling Facilitates Amygdala-Anterior Cingulate Cortex Synchrony and Decision Making in Rats. Cell Rep..

[11] Icenhour A., Langhorst J., Benson S., Schlamann M., Hampel S., Engler H., Forsting M., Elsenbruch S. (2015). Neural circuitry of abdominal pain-related fear learning and reinstatement in irritable bowel syndrome. Neurogastroenterol. Motil..

[12] Mayer E.A., Naliboff B.D., Craig A.D. (2006). Neuroimaging of the brain-gut axis: From basic understanding to treatment of functional GI disorders. Gastroenterology.

[13] Bushnell M.C., Ceko M., Low L.A. (2013). Cognitive and emotional control of pain and its disruption in chronic pain. Nat. Rev. Neurosci..

[14] Woodworth R.S., Sherrington C.S. (1904). A pseudaffective reflex and its spinal path. J. Physiol..

[15] Cao B., Zhang X., Yan N., Chen S., Li Y. (2012). Cholecystokinin enhances visceral pain-related affective memory via vagal afferent pathway in rats. Mol. Brain.

[16] Yan N., Cao B., Xu J., Hao C., Zhang X., Li Y. (2012). Glutamatergic activation of anterior cingulate cortex mediates the affective component of visceral pain memory in rats. Neurobiol. Learn. Mem..

[17] Bezzi P., Volterra A. (2011). Astrocytes: Powering memory. Cell.

[18] Kol A., Adamsky A., Groysman M., Kreisel T., London M., Goshen I. (2020). Astrocytes contribute to remote memory formation by modulating hippocampal-cortical communication during learning. Nat. Neurosci..

[19] Suzuki A., Stern S.A., Bozdagi O., Huntley G.W., Walker R.H., Magistretti P.J., Cristina M.A. (2011). Astrocyte-neuron lactate transport is required for long-term memory formation. Cell.

[20] Verkhratsky A., Nedergaard M. (2018). Physiology of Astroglia. Physiol. Rev..

[21] Pellerin L., Magistretti P.J. (1994). Glutamate uptake into astrocytes stimulates aerobic glycolysis: A mechanism coupling neuronal activity to glucose utilization. Proc. Natl. Acad. Sci. USA.

[22] Hu Y., Wilson G.S. (1997). A temporary local energy pool coupled to neuronal activity: Fluctuations of extracellular lactate levels in rat brain monitored with rapid-response enzyme-based sensor. J. Neurochem..

[23] Prichard J., Rothman D., Novotny E., Petroff O., Kuwabara T., Avison M., Howseman A., Hanstock C., Shulman R. (1991). Lactate rise detected by 1H NMR in human visual cortex during physiologic stimulation. Proc. Natl. Acad. Sci. USA.

[24] Gibbs M.E., Anderson D.G., Hertz L. (2006). Inhibition of glycogenolysis in astrocytes interrupts memory consolidation in young chickens. Glia.

[25] Walls A.B., Sickmann H.M., Brown A., Bouman S.D., Ransom B., Schousboe A., Waagepetersen H.S. (2008). Characterization of 1,4-dideoxy-1,4-imino-d-arabinitol (DAB) as an inhibitor of brain glycogen shunt activity. J. Neurochem..

[26] Adamsky A., Kol A., Kreisel T., Doron A., Ozeri-Engelhard N., Melcer T., Refaeli R., Horn H., Regev L., Groysman M. (2018). Astrocytic Activation Generates De Novo Neuronal Potentiation and Memory Enhancement. Cell.

[27] Johansen J.P., Fields H.L., Manning B.H. (2001). The affective component of pain in rodents: Direct evidence for a contribution of the anterior cingulate cortex. Proc. Natl. Acad. Sci. USA.

[28] Llorca-Torralba M., Suárez-Pereira I., Bravo L., Camarena-Delgado C., Garcia-Partida J.A., Mico J.A., Berrocoso E. (2019). Chemogenetic Silencing of the Locus Coeruleus-Basolateral Amygdala Pathway Abolishes Pain-Induced Anxiety and Enhanced Aversive Learning in Rats. Biol. Psychiatry.

[29] Wu X., Gao J., Yan J., Fan J., Owyang C., Li Y. (2008). Role for NMDA receptors in visceral nociceptive transmission in the anterior cingulate cortex of viscerally hypersensitive rats. Am. J. Physiol. Gastrointest. Liver Physiol..

[30] Sofroniew M.V., Vinters H.V. (2010). Astrocytes: Biology and pathology. Acta Neuropathol..

[31] Santello M., Toni N., Volterra A. (2019). Astrocyte function from information processing to cognition and cognitive impairment. Nat. Neurosci..

[32] Attwell D., Laughlin S.B. (2001). An energy budget for signaling in the grey matter of the brain. J. Cereb. Blood Flow Metab..

[33] Tang F., Lane S., Korsak A., Paton J.F.R., Gourine A.V., Kasparov S., Teschemacher A.G. (2014). Lactate-mediated glia-neuronal signalling in the mammalian brain. Nat. Commun..

[34] Cataldo A.M., Broadwell R.D. (1986). Cytochemical identification of cerebral glycogen and glucose-6-phosphatase activity under normal and experimental conditions. II. Choroid plexus and ependymal epithelia, endothelia and pericytes. J. Neurocytol..

[35] Dringen R., Gebhardt R., Hamprecht B. (1993). Glycogen in astrocytes: Possible function as lactate supply for neighboring cells. Brain Res..

[36] Shulman R.G., Hyder F., Rothman D.L. (2001). Cerebral energetics and the glycogen shunt: Neurochemical basis of functional imaging. Proc. Natl. Acad. Sci. USA.

[37] Rouach N., Koulakoff A., Abudara V., Willecke K., Giaume C. (2008). Astroglial metabolic networks sustain hippocampal synaptic transmission. Science.

[38] Magistretti P.J., Allaman I. (2018). Lactate in the brain: From metabolic end-product to signalling molecule. Nat. Rev. Neurosci..

[39] Lauritzen K.H., Morland C., Puchades M., Holm-Hansen S., Hagelin E.M., Lauritzen F., Attramadal H., Storm-Mathisen J., Gjedde A., Bergersen L.H. (2014). Lactate receptor sites link neurotransmission, neurovascular coupling, and brain energy metabolism. Cereb. Cortex.

[40] Morland C., Lauritzen K.H., Puchades M., Holm-Hansen S., Andersson K., Gjedde A., Attramadal H., Storm-Mathisen J., Bergersen L.H. (2015). The lactate receptor, G-protein coupled receptor81/hydroxycarboxylic acid receptor 1: Expression and action in brain. J. Neurosci. Res..

[41] Briquet M., Rocher A.B., Alessandri M., Rosenberg N., de Castro Abrantes H., Wellbourne-Wood J., Schmuziger C., Ginet V., Puyal J., Pralong E. (2022). Activation of lactate receptor HCAR1 down-modulates neuronal activity in rodent and human brain tissue. J. Cereb. Blood Flow Metab..

[42] De Castro Abrantes H., Briquet M., Schmuziger C., Restivo L., Puyal J., Rosenberg N., Rocher A.B., Offermanns S., Chatton J.V. (2019). The lactate receptor HCAR1 modulates neuronal network activity through the activation of Gα and Gβγ subunits. J. Neurosci..

[43] Wallenius K., Thalen P., Bjorkman J.A., Petra Johannesson P., Wiseman J., Böttcher G., Fjellström O., Oakes N.D. (2017). Involvement of the metabolic sensor GPR81 in cardiovascular control. JCI Insight.

[44] Yang J., Ruchti E., Petit J.M., Jourdain P., Grenningloh G., Allaman I., Magistretti P.J. (2014). Lactate promotes plasticity gene expression by potentiating NMDA signaling in neurons. Proc. Natl. Acad. Sci. USA.

[45] Mosienko V., Teschemacher A.G., Kasparov S. (2015). Is L-lactate a novel signaling molecule in the brain?. J. Cereb. Blood Flow Metab..

